# Gaussian noise up-sampling is better suited than SMOTE and ADASYN for clinical decision making

**DOI:** 10.1186/s13040-021-00283-6

**Published:** 2021-11-29

**Authors:** Jacqueline Beinecke, Dominik Heider

**Affiliations:** Department of Mathematics and Computer Science, Philipps-University of Marburg, Hans-Meerwein-Str. 6, 35043 Marburg, Germany

**Keywords:** Machine learning, Clinical data, Data augmentation, Synthetic data

## Abstract

Clinical data sets have very special properties and suffer from many caveats in machine learning. They typically show a high-class imbalance, have a small number of samples and a large number of parameters, and have missing values. While feature selection approaches and imputation techniques address the former problems, the class imbalance is typically addressed using augmentation techniques. However, these techniques have been developed for big data analytics, and their suitability for clinical data sets is unclear.

This study analyzed different augmentation techniques for use in clinical data sets and subsequent employment of machine learning-based classification. It turns out that Gaussian Noise Up-Sampling (GNUS) is not always but generally, is as good as SMOTE and ADASYN and even outperform those on some datasets. However, it has also been shown that augmentation does not improve classification at all in some cases.

## Introduction

Machine learning (ML) and artificial intelligence (AI) have entered many areas of life and will also pave the way to a new era in medicine. These models can improve medical treatment or diagnosis, identify novel subtypes, or give new insights into survival prognostics. These models consider all facets of data types, e.g., clinical health records, image, or omics data. Clinical decision-support-systems based on ML and AI have been successfully used in many different studies and medical fields, e.g., oncology [1], pathology [2–4], diabetes [5, 6], human genetics [7], and infectious diseases [8–10] as part of a growing trend toward personalized / precision medicine.

Overall, there is great potential for clinical decision-support-systems based on ML and AI techniques. However, clinical datasets have very special properties and suffer from many caveats regarding ML and AI, e.g., a high class imbalance. Moreover, clinical decision-support-systems in medicine need to be interpretable in a probabilistic manner, typically addressed by calibration methods [11]. Furthermore, small-n-large-p and missing values are addressed by feature selection (also called biomarker discovery) approaches [12] and imputation techniques [13]. The class imbalance is typically addressed by using down-sampling or data augmentation techniques [37|. ML and AI approaches perform worse when the data is imbalanced, i.e., when the proportion of positive samples (i.e., cases) and negative samples (i.e., controls) differ strongly. The resulting model will be biased towards the majority class [14]. This is frequently found in clinical datasets, e.g., for rare diseases, but also for general cohort data. While down-sampling might be a straightforward approach to balance a dataset, it is typically worse than data augmentation techniques, as important information / associations from the majority class might be lost. Moreover, down-sampling is not an option when the dataset is already relatively small, as it is often the case in clinical settings.

Methods for addressing the class imbalance by data augmentation have been developed for big data analytics. However, their suitability for clinical data sets has not been tested yet and remains unclear. In the current study, we analyzed commonly used data augmentation techniques in several imbalanced clinical datasets and different ML models.

## Methods

### Data

In our study, we used ten clinical datasets covering different diseases/scenarios from different medical fields, such as oncology, reproduction, psychology, or hepatology. These datasets address breast cancer, cervical cancer, fertility, drug abuse, hepatitis, cardiotocography, and fatty liver disease, to reflect different sample sizes and class imbalances. Nine of these datasets were collected from the UCI Machine Learning Repository [15]. The smallest dataset has 72 samples, and the most extensive dataset consists of 1831 samples. On average, the datasets have 540 samples, the median is 426, 1st and 3rd quartiles are 124.5 and 713, respectively. The imbalance differs between 2.23 and 37.26% concerning the cases (i.e., positive class). On average, the imbalance is 18.42% (median is 17.7%), with 1st and 3rd quartile at 9.78 and 28.49%, respectively. The number of features ranges from 3 to 32, on average 15 (median is 11), with 1st and 3rd quartile of 9 and 21, respectively.

An overview of the datasets can be found in Table 1. We removed all samples and features with missing values. Thus, the numbers may differ slightly from the original number of samples and features.

**Table 1:**
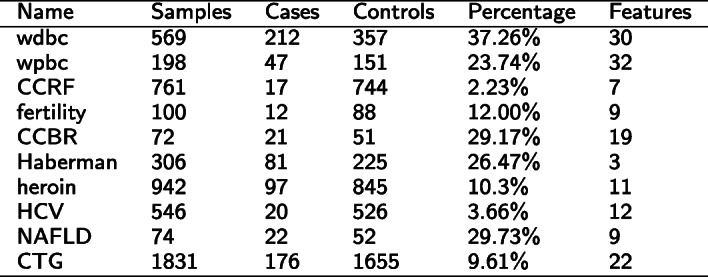
Overview of the datasets

The Breast Cancer diagnostics dataset (wdbc) consists of 569 breast cancer patients [357 benign, 212 (37.26%) malignant] with 30 attributes describing histological cancer characteristics [16].

The Breast Cancer prognostics dataset (wpbc) consists of 198 breast cancer patients [151 non-recur, 47 (23.74%) recur] with 32 attributes [16].

The Haberman’s survival dataset captures three numerical clinical characteristics and the survival status (binary) for 306 breast cancer patients [225 (73.5%; referred to as controls) survived 5 years or longer, 81 (26.5%; referred to as cases) died within 5 years] [17]. The models predict the survival status (case/control) as a function of three numerical features, namely the patient’s age at the time of surgery, the patient’s year of surgery, and the number of positive axillary nodes detected.

We used two datasets for cervical cancer, namely the Cervical cancer (Risk Factors) Data Set (CCRF) and the Cervical Cancer Behavior Risk Data Set (CCBR).

The original CCRF dataset comprises demographic information, habits, and historical medical records of 858 patients [18]. We removed all samples and features with missing values. Thus, the final CCRF dataset consists of 761 samples [744 controls and 17 cases (2.23%)] with 7 features. The CCBR dataset consists of 72 samples [51 controls and 21 cases (29.17%)] with 19 features [19].

The fertility dataset consists of 100 samples [88 controls and 12 cases (12.00%)] with 9 features, such as age, childish diseases, etc., and can be used to predict seminal quality [20].

The heroin dataset is a subset of the Drug consumption dataset [21], consisting of data from different drug users. For our analyses, we used just a subset, namely the female heroin abusers. The heroin dataset consists of 942 samples [845 non-heroin abusers and 97 heroin abusers (10.30%)] with 11 attributes, including age, different personality measurements, etc.

The HCV dataset contains laboratory values of blood donors and Hepatitis C patients as well as demographic values and consists of 546 samples [526 controls and 20 cases (3.66%)] [22].

The NAFLD dataset consists of 74 patients with fatty liver disease. Fifty two of them have a non-alcoholic fatty liver, while 22 have an alcoholic fatty liver (29.73%). The dataset has 9 features, including demographic values as well as blood parameters [23].

The Cardiotocography dataset (CTG) consists of 1831 fetal cardiotocograms, of which 1655 are normal and 176 have been classified as pathologic (9.61%). The dataset provides 22 features that have been calculated based on the cardiotocograms [24].

### Augmentation techniques

An imbalance is frequently found in real-world datasets, in particular in biomedical datasets. Two main approaches can be found in the literature used to rebalance the data prior to machine learning modeling, namely under-sampling and up-sampling. In under-sampling, we downsize the actual dataset so that the ratio of the dependent categories is balanced. However, due to the nature of clinical data, which is typically relatively small, under-sampling is not an appropriate approach. Thus, our study focuses only on up-sampling techniques (also referred to as data augmentation).

We analyzed three frequently used approaches for data augmentation, namely SMOTE, ADASYN, and GNUS, and compared them to the model without any data augmentation (from now on referred to as the null model) and with each other.

SMOTE (Synthetic Minority Over-sampling Technique) is based on the k-nearest neighbor algorithm [25]. First, it finds the k-nearest neighbors in the minority class for each of the samples in the class. Then it draws a line between the neighbors and generates random points on the lines. We used SMOTE with default settings, i.e., the number of k-nearest neighbors is set to 5.

ADASYN (Adaptive Synthetic sampling approach) works similar to SMOTE and generates synthetic observations for the minority class [26]. However, it adds some noise and thus introduces some variance to the synthetic data points generated from the k-nearest neighbors. We used ADASYN with default settings, i.e., the number of k-nearest neighbors is set to 5. We used the R package *smotefamily* v.1.3.1 for SMOTE and ADASYN.

GNUS (Gaussian Noise Up-Sampling) is a very straightforward and fast technique to generate synthetic data points. It is based on up-sampling [27], i.e., it randomly selects samples from the minority class and adds them to the training data. However, in contrast to normal up-sampling, GNUS adds some noise to the synthetic data points improving variance and smoothing the class boundary to reduce overfitting. Several types of noise could be used. However, the most common one is Gaussian noise. We used GNUS with normal distribution with $\overline{x}={\overline{x}}_i\ast 0.001$ and *sd* = *sd*_*i*_ ∗ 0.001 for *i ϵ* 1, …, *n*, and *n* the number of features (i.e., columns) in the dataset.

### Machine learning models

To compare the different augmentation techniques for clinical datasets on subsequent classification, we used three different statistical and machine learning algorithms that are frequently used in clinical settings, namely Logistic Regression (LR), Support Vector Machines (SVM), and Random Forests (RF). For the SVMs, we employed different kernels to capture linear and non-linear associations, namely the linear, radial basis function (rbf), and polynomial kernels. We used the R packages *randomforest* v.4.6 and *kernlab* v.0.9 [28], to train the RF and SVMs, respectively. The LR was trained with the *glm* function in R using the logit model. RFs and SVMs were trained with default parameters.

### Statistical evaluation

The statistical and machine learning models were trained and evaluated based on 1000 times repeated Monte Carlo cross-validation (MCCV) [29] using the area under the receiver operating characteristics curve (AUC), area under the precision-recall curve (PR), F1, and the Matthews correlation coefficient (MCC) calculated using the R package *ROCR* v.1.0 [30]. Due to the nature of the data, we used MCCV (also referred to as repeated random sub-sampling validation) instead of the leave-one-out CV, according to Xu et al. [31]. The MCCV has a more minor variance and thus is more reliable for comparison in small datasets. However, it has a higher bias than the k-fold CV. For large sample sizes, the variance issues become less important. However, the datasets are relatively small, and bias does not play a decisive role but variance, as we do not aim at having the best model, but instead want to compare different augmentation techniques with each other. The Bias-Variance trade-off is very common in machine learning, however, in our study, a low variance is more important than a low bias.

The significance of the differences was calculated based on Student’s t-tests, resulting *p* values were corrected for multiple testing by the method of Benjamini and Hochberg [32].

## Results

**Fig. 1:**
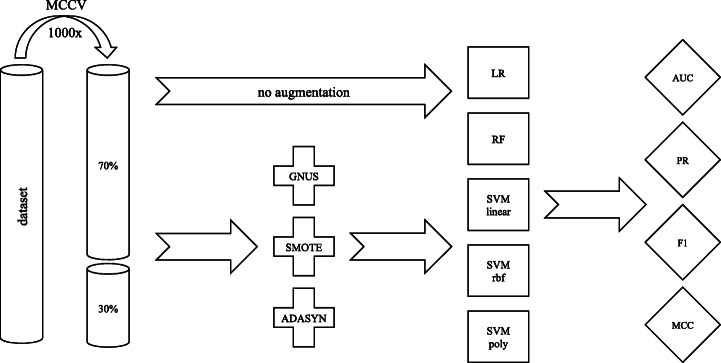
The workflow of the study. Models are evaluated with 1000 times repeated MCCV. Three augmentation techniques, namely GNUS, SMOTE, and ADASYN are compared against each other and to the null model. As machine learning models we used logistic regression (LR), random forests (RF), and support-vector machines (SVM) with three different kernels (linear, rbf, and polynomial). As for performance metrics, we used AUC, PR, F1, and MCC

The workflow of the current study is shown in Fig. 1.

**Fig. 2:**
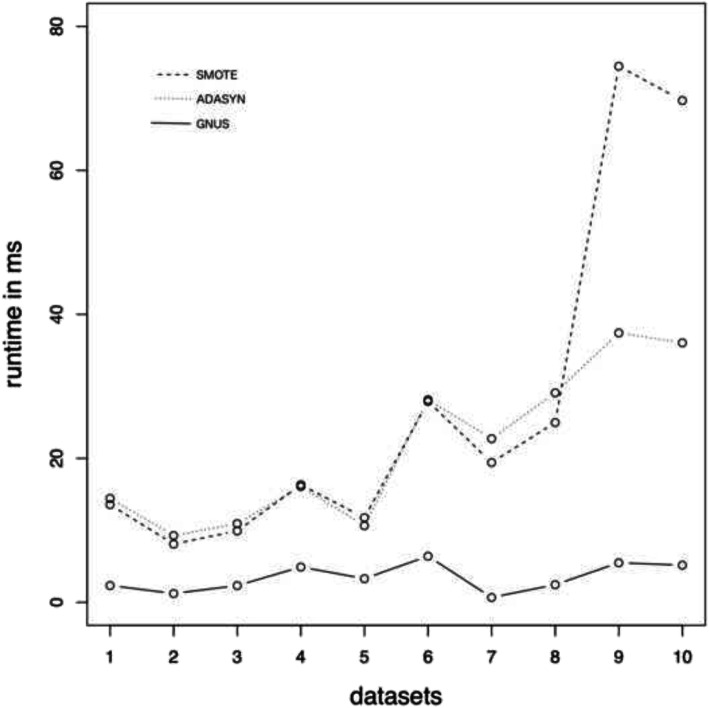
The runtime of GNUS, SMOTE, and ADASYN on the datasets. GNUS is shown as a solid line, SMOTE is shown as a dashed line, and ADASYN is shown as a dotted line. Datasets are sorted according to the number of cases in the dataset. 1:fertility, 2:CCRF, 3:HCV, 4:CCBR, 5:NAFLD, 6:wpbc, 7:Haberman, 8:heroin, 9:ctg, 10:wdbc

GNUS shows significantly (*p* < 0.001 for all datasets) smaller runtime compared to SMOTE and ADASYN (see Fig. 2). On average, GNUS is 9.41 times (*p* < 0.001) faster than SMOTE and 8.9 times (*p* = 0.009) faster than ADASYN, while SMOTE and ADASYN show no significant differences in runtime (*p* = 0.2423). The runtime of GNUS significantly correlates with the number of features (*r* = 0.9347, *p* < 0.001), while SMOTE and ADASYN show only significant correlation with the number of cases in the datasets (*r* = 0.9468, *p* < 0.001 and *r* = 0.8996, *p* < 0.001).

The runtime was measured on a MacBook Pro with 3.5 GHz Dual-Core Intel Core i7 with 16 GB 2133 MHz LPDDR3 RAM.

Data augmentation with neither GNUS, SMOTE, or ADASYN improved subsequent classification with any of the tested statistical and machine learning models significantly for the NAFLD dataset.

For the other nine datasets, data augmentation could improve subsequent classification significantly. An overview of the nine datasets’ AUC and MCC values are shown in Tables 2 and 3, as a representative selection of metrics [33].

**Table 2:**
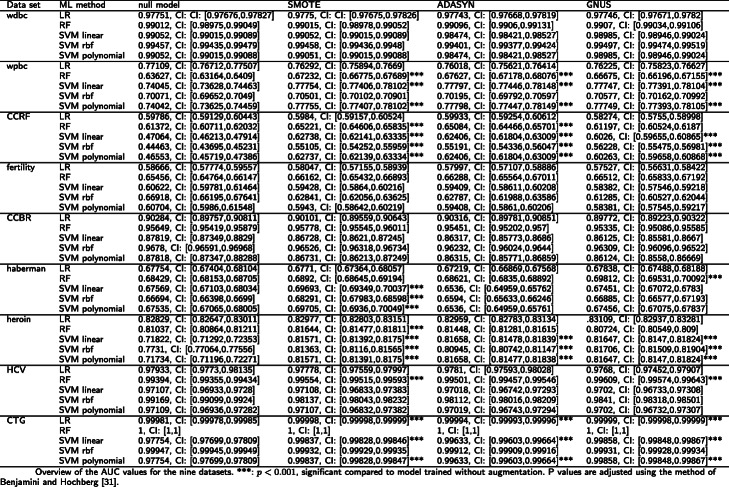
Overview of the AUC values for the nine datasets. ***: *p* < 0.001, significant compared to model trained without augmentation. *P* values are adjusted using the method of Benjamini and Hochberg [32]

**Table 3:**
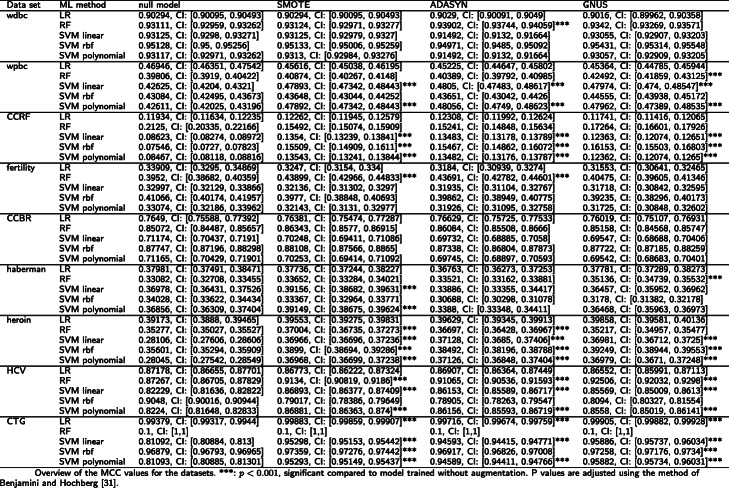
Overview of the MCC values for the nine datasets. ***: *p* < 0.001, significant compared to model trained without augmentation. P values are adjusted using the method of Benjamini and Hochberg [32]

For the CCBR dataset, SMOTE improved subsequent classification in terms of F1 for the RF from 0.88921 to 0.90024 with *p* < 0.001. ADASYN and GNUS were not able to improve predictions.

All data augmentation methods improved PR, MCC, and F1 for the RF on the fertility dataset. However, for GNUS, the differences were not significant. SMOTE increased MCC from 0.3952 to 0.43899 with *p* < 0.001 (ADASYN: *MCC* = 0.43691 with *p* < 0.001, GNUS: *MCC* = 0.40475 with *p* > 0.001). All data augmentation methods increased AUC for the RF, but no difference was significant.

For the wpbc dataset, all data augmentation methods improved subsequent classification in terms of all metrics for all SVMs and the RF. However, not all differences were significant. All data augmentation methods significantly improved (*p* < 0.001) all metrics for the SVM with linear and polynomial kernel. For the RF, all augmentation methods significantly increased (*p* < 0.001) AUC and F1. Furthermore, GNUS and SMOTE increased the PR for the RF significantly (*p* < 0.001). GNUS significantly increased (*p* < 0.001) MCC for RF from 0.39806 to 0.42492, which was significantly larger than both SMOTE and ADASYN (SMOTE: *MCC* = 0.40874, ADASYN: *MCC* = 0.0.40389). Moreover, GNUS performed significantly better than ADASYN for the RF in terms of PR. In addition, GNUS significantly increased (*p* < 0.001) F1 for SVM with rbf kernel from 0.51902 to 0.53424. A significant decrease for the LR in terms of PR was achieved by all data augmentation methods, AUC and F1 by ADASYN, and MCC by ADASYN and GNUS.

On the Haberman dataset, all data augmentation methods significantly increased F1 for the RF, but in this case, GNUS performed significantly better than SMOTE and ADASYN. GNUS increased F1 for the RF from 0.52521 to 0.54206 (SMOTE: F1 = 0.53165, ADASYN: F1 = 0.53117). Besides that, all data augmentation methods improved the RF in terms of AUC and MCC, but the differences were only significant for GNU, which also performed significantly better than ADASYN and SMOTE in this case. SMOTE was able to improve subsequent classification in terms of all metrics for the SVM with linear and polynomial kernel, as well as AUC for the SVM with rbf kernel. For the SVM with the linear and the polynomial kernel in terms of PR, SMOTE performed significantly better than GNUS, and for the SVM with rbf kernel in terms of AUC.

For the HCV dataset, all data augmentation methods were able to improve PR, MCC, and F1 for SVM with linear and polynomial kernel and for the RF. Furthermore, all augmentation methods increased AUC for the RF, but only GNUS and SMOTE significantly. For the RF in terms of all metrics, GNUS performed significantly better than ADASYN and for the SVM with linear and polynomial kernel SMOTE performed significantly better than GNUS in terms of PR. GNUS increased AUC for the RF from 0.99394 to 0.99609 with *p* < 0.001 (SMOTE: AUC = 0.99554 with *p* < 0.001, ADASYN: AUC = 0.99501).

ADASYN significantly increased PR, MCC, and F1 for the RF on the wdbc dataset. For MCC and F1 in terms of the RF ADASYN performed significantly better than both GNUS and SMOTE. For instance, ADASYN increased F1 from 0.95607 to 0.96095 with *p* < 0.001 (SMOTE: F1 = 0.95621, GNUS: F1 = 0.958). Furthermore, ADASYN significantly decreased all metrics for the SVM with linear and polynomial kernel, and PR for the SVM with rbf kernel. GNUS and SMOTE were able to increase all metrics for the SVM with rbf kernel, but only GNUS significantly for PR. GNUS performed significantly better than SMOTE.

For the CCRF dataset, all data augmentation methods significantly improved subsequent classification in terms of all metrics for all SVMs. For example, SMOTE increased AUC for the SVM with linear kernel from 0.47064 to 0.62738 (ADASYN: AUC = 0.62406, GNUS: AUC = 0.6026). In terms of AUC, MCC, and F1 for SVM with linear and polynomial kernel ADASYN and SMOTE performed significantly better than GNUS. Furthermore, ADASYN and SMOTE significantly increased AUC for the RF. Lastly, SMOTE and ADASYN increased AUC, PR, MCC, and F1 for the LR and GNUS increased PR and F1 for the LR, but no significant increase. No data augmentation method was able to improve PR, MCC, or F1 for the RF.

All data augmentation methods were able to improve all metrics for the SVMs on the heroin dataset. All differences were significant except for the SVM with rbf kernel in terms of PR. Furthermore, SMOTE and ADASYN increased AUC, MCC, and F1 for the LR and the RF, with significant differences for SMOTE in terms of AUC, MCC, and F1 for the RF, and for ADASYN in terms of MCC and F1 for the RF. In addition, SMOTE and ADASYN performed significantly better than GNUS for the RF in terms F1. In contrast, GNUS performed significantly better than ADASYN for the SVM with rbf kernel in terms of AUC. Lastly, GNUS improved the LR in terms of AUC, MCC, and F1 and the RF in terms of F1, but none of the differences were significant.

For the CTG dataset, all data augmentation methods significantly improved subsequent classification in terms of all metrics for the LR and SVM with the linear and polynomial kernel. For the SVM with rbf kernel all data augmentation methods increased MCC and F1, however, only significantly for SMOTE and GNUS. Furthermore, for the SVM with linear and polynomial kernel in terms of MCC, PR, and F1 GNUS performed significantly better than ADASYN and SMOTE, and SMOTE performed significantly better than ADASYN. Additionally, for the LR in terms of all metrics SMOTE and GNUS performed significantly better than ADASYN. This also holds for the SVM with linear and polynomial kernel in terms of AUC, and the SVM with rbf kernel in terms of MCC and F1.

For the three smallest datasets, namely CCBR (72 samples), NAFLD (74 samples), and fertility (100 samples), data augmentation did not improve subsequent classification a lot. This is also true for the wdbc dataset, which is larger (569 samples) but is the least imbalanced (37.26%) out of all of the ten datasets.

The wpbc and Haberman datasets are the next larger ones with 198 samples and 306 samples. Both are similarly imbalanced (wdbc = 23.74% and Haberman = 26.47%) but the Haberman dataset only has three features while the wpbc dataset has 32. This could be why the Haberman dataset profited significantly less from the data augmentation methods than the wpbc dataset.

The HCV dataset (546 samples and 3.66%), CCRF dataset (761 and 2.23%), heroin dataset (942 samples and 10.3%), and the CTG dataset (1831 samples and 9.61%) are the largest and most imbalanced datasets, and next to the wpbc dataset they profited the most from all data augmentation methods.

## Discussion

Data augmentation techniques have been developed to tackle the problem of class imbalance and subsequent bias in machine learning models. However, these methods have been developed for big data analytics, and their applicability and suitability for clinical data sets, which are relatively small, have not been evaluated widely so far.

Taneja et al. compared SMOTE and ADASYN with RFs and gradient boosting approaches on a single dataset (with 284,807 samples) with a high degree of imbalance [34]. Their results show that the metrics from all models in conjunction with SMOTE outperformed ADASYN. Another analysis on a single data set (7718 samples) with a high degree of imbalance was carried out by Barros et al. [35]. They compared SMOTE and ADASYN based on subsequent classification with decision trees and neural networks and could also show that SMOTE outperforms ADASYN on this dataset. Davagdorj et al. compared SMOTE and ADASYN based on seven different statistical and machine learning models on a single dataset (3692 samples). However, the performance of SMOTE and ADASYN varied depending on the model [36]. Amin et al. included four datasets (between 3333 and 38,162 samples) with varying degrees of imbalance and four different machine learning models [37]. However, neither SMOTE nor ADASYN were superior to each other. To the best of our knowledge, our study marks the first comparison of SMOTE, ADASYN, and GNUS on a larger number of datasets and models and the first study that addresses clinical data and clinical decision making.

For some datasets, data augmentation did not improve overall performance in subsequent machine learning. It turned out that for small datasets (≤100 samples), data augmentation is less useful for subsequent classification than for larger datasets. For larger datasets (≥546 samples) with a high-class imbalance (≤10.3%), all three data augmentation methods significantly improved performance in subsequent classification.

In most datasets, GNUS is as good as SMOTE or ADASYN, and a significant improvement has been reached compared to the models without data augmentation. In line with some other studies, SMOTE generally performs better than ADASYN. There are, however, also some cases where GNUS is significantly better than SMOTE or ADASYN, e.g., in the wdbc dataset. Moreover, in some cases, data augmentation significantly decreased performance concerning compared to the imbalanced model. In all datasets analyzed, GNUS significantly outperforms SMOTE and ADASYN in terms of runtime.

Nevertheless, data augmentation can only help to decrease bias in imbalanced datasets for machine learning predictions. If the data has not enough variance, data augmentation will not improve subsequent predictions. In the worst case, can also decrease performance because of the additional noise introduced in the training of the models.

This study marks the first comprehensive analysis of three commonly used data augmentation techniques for use in clinical datasets with a variety of machine learning models. It turned out that simple GNUS is generally as good as SMOTE and ADASYN and on some datasets and models even outperformed them.

## Data Availability

All datasets are publicly available at the UCI Machine Learning Repository.
